# Programmed Fetal Membrane Senescence and Exosome-Mediated Signaling: A Mechanism Associated With Timing of Human Parturition

**DOI:** 10.3389/fendo.2017.00196

**Published:** 2017-08-17

**Authors:** Ramkumar Menon, Sam Mesiano, Robert N. Taylor

**Affiliations:** ^1^Department of Obstetrics and Gynecology, Division of Maternal-Fetal Medicine Perinatal Research, The University of Texas Medical Branch, Galveston, TX, United States; ^2^Department of Reproductive Biology and Obstetrics and Gynecology, Case Western Reserve University, Cleveland, OH, United States; ^3^Department of Obstetrics and Gynecology, Wake Forest School of Medicine, Winston-Salem, NC, United States

**Keywords:** fetal signals, parturition, exosomes, biomarker, aging, fetal membranes, p38MAPK, microvesicles

## Abstract

Human parturition is an inflammatory process that involves both fetal and maternal compartments. The precise immune cell interactions have not been well delineated in human uterine tissues during parturition, but insights into human labor initiation have been informed by studies in animal models. Unfortunately, the timing of parturition relative to fetal maturation varies among viviparous species—indicative of different phylogenetic clocks and alarms—but what is clear is that important common pathways must converge to control the birth process. Herein, we hypothesize a novel signaling mechanism initiated by human fetal membrane aging and senescence-associated inflammation. Programmed events of fetal membrane aging coincide with fetal growth and organ maturation. Mechanistically, senescence involves in telomere shortening and activation of p38 mitogen-activated signaling kinase resulting in aging-associated phenotypic transition. Senescent tissues release inflammatory signals that are propagated *via* exosomes to cause functional changes in maternal uterine tissues. *In vitro*, oxidative stress causes increased release of inflammatory mediators (senescence-associated secretory phenotype and damage-associated molecular pattern markers) that can be packaged inside the exosomes. These exosomes traverse through tissues layers, reach maternal tissues to increase overall inflammatory load transitioning them from a quiescent to active state. Animal model studies have shown that fetal exosomes can travel from fetal to the maternal side. Thus, aging fetal membranes and membrane-derived exosomes cargo fetal signals to the uterus and cervix and may trigger parturition. This review highlights a novel hypothesis in human parturition research based on data from ongoing research using human fetal membrane model system.

Preterm birth (delivery before the 37th week of gestation) has increased globally nearly 30% in the last 25 years despite improvements in perinatal care (1). To address PTB, a clear understanding of the signals that initiate labor is needed (2). Both preterm, specifically spontaneous preterm, and term parturition share common terminal pathways that involve intrauterine inflammation and oxidative stress (OS), resulting in myometrial contractions and cervical remodeling (2, 3). Significant knowledge gaps exist, but over the past decade, we have come to realize that fetal endocrine signals, particularly those derived from the adrenal axis (i.e., CRH, ACTH) function as a biologic clock, concomitantly triggering organ maturation and labor and delivery at term (4). We hypothesize, however, that endocrine signals alone are not sufficient to disrupt the homeostatic balance that maintains uterine quiescence. Inflammation and OS of the amniochorionic (fetal) membranes at the feto-maternal interface are postulated as signals that perturb the relaxed uterine state (3). In this brief review, we introduce the concept and provide circumstantial evidence that *in utero* aging of the fetal membranes generates inflammatory proteins and prostanoids that trigger parturition.

## Aging

**Figure 1:**
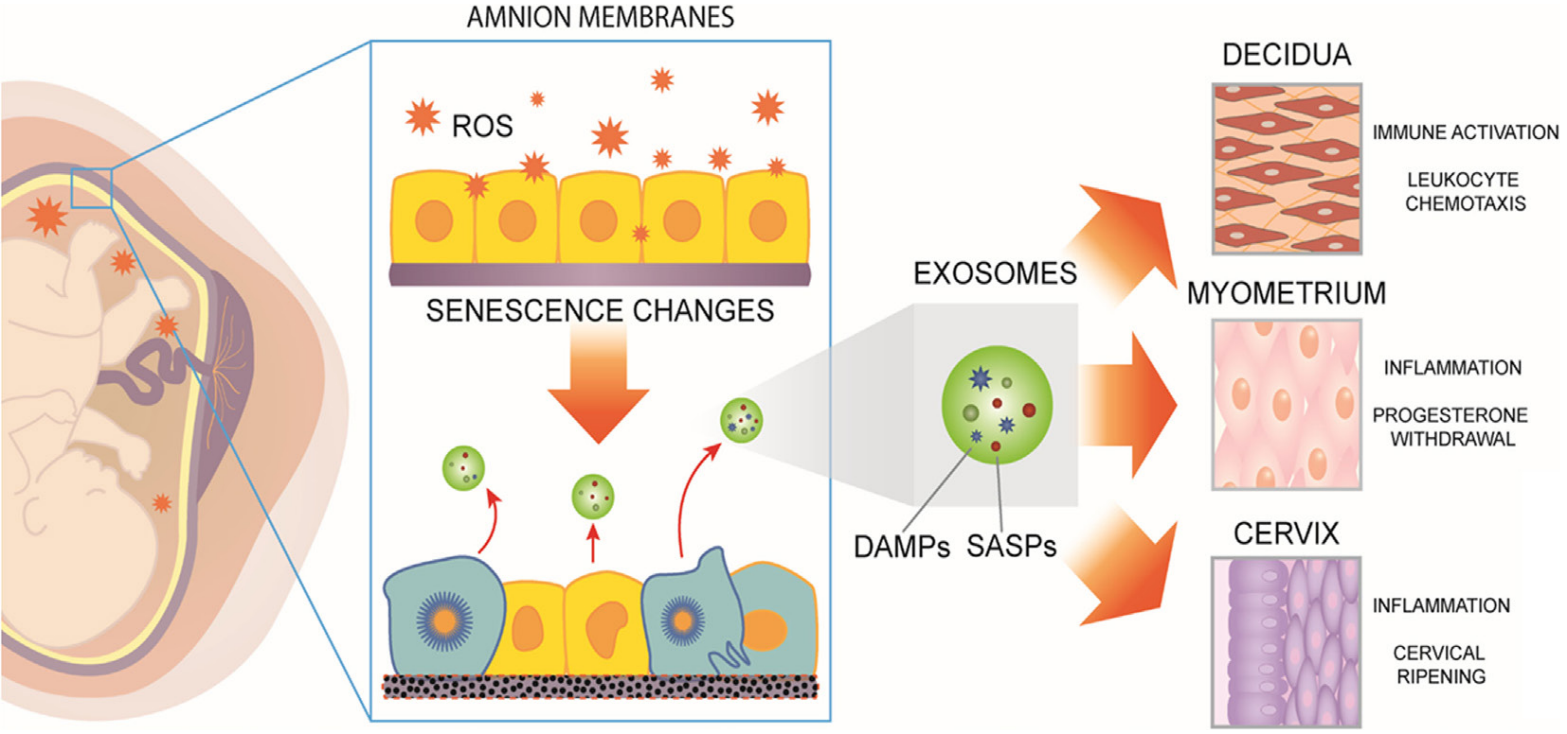
Human fetal membranes undergo cumulative oxidative stress during gestation. Reactive oxygen species (ROS) can lead to telomere-dependent, p38-mediated amnion cell senescence. Aging within fetal membranes coincides with fetal growth and organ maturation and, therefore, senescence-associated molecular signals can be hypothesized as proxies for fetal parturition signals. Senescent fetal cells release signals in the form of senescence-associated secretory phenotype and damage-associated molecular pattern (DAMP) markers [senescence-associated secretory proteins (SASPs) and DAMPs]. They can be collectively considered as sterile inflammatory mediators of parturition. SASPs and DAMPs can be packaged inside exosomes and propagated to maternal uterine tissues. In decidua, myometrium, and cervix, fetal-derived exosomes fuse with maternal target cells and deliver their cargo, increasing a localized inflammatory load. When inflammation reaches a threshold, quiescent uterine tissues transition to an active laboring state. Thus, fetal exosomes serve as signals of fetal readiness for parturition. In summary, fetal tissue derived exosomes that can be isolated from maternal blood could serve as biomarkers of fetal maturation at term. In preterm labor, fetal exosome cargo content may reflect pathophysiologic derangements and serve as a biomarker indicative of imminent delivery.

The amniochorionic membranes surround the intrauterine cavity, providing a structural barrier to contain amniotic fluid for the growing fetus (5). The aging of these membranes is now recognized as a contributor of labor inducing signals (6). Like all tissues, the fetal membranes undergo aging, a natural, biologic phenomenon of random stochastic changes that result in altered molecular structure and function. Aging *in utero* is associated with the development of the fetus, with acceleration of this process projected to have long-term programming consequences in later life, potentially predisposing to adult-onset diseases. We posit that aging of the fetal membranes developmentally synchronize with the fetus to dictate the duration of pregnancy. As we have reported, progressive reductions in telomere length of fetal membrane cell chromosomes parallel those in fetal leukocytes (7). Herein, we hypothesize that fetal membrane aging is associated with sterile inflammatory changes (Figure 1) propagated *via* exosomes (30–100 nm spherical microvesicles) from amniochorionic cells to maternal tissues. Accelerated fetal membrane aging, manifested as telomere length reduction, is influenced by biochemical mediators of OS generated within fetal organs as they mature (8). OS fluctuates throughout pregnancy and maximum OS is seen at term (9–17). This is partly due to increased metabolic demand from the fetus, reduced maternal supply of substrate, no change in antioxidant status in uterine tissues creating an imbalance in redox state and increased stretching of membranes. This increased OS accelerates telomere attrition as Guanine nucleotide base-rich telomere are highly susceptible to OS induced DNA damage.

## Mechanisms of Fetal Membrane Aging

Aging is an inevitable process in the life of all cells and organisms (18). OS plays a major role in regulating aging process (19, 20), especially in intrauterine tissues during pregnancy (21–23). OS builds up in part by an increase in fetal energy utilization as gestation progresses, but also due to the limited maternal supply of metabolic substrates and low antioxidant reserves (11). The peroxidation of cellular proteins, lipids, and DNA activate the p38 mitogen-activated protein kinase (MAPK) signaling pathway (24, 25), a pluripotent stress mediator that induces p16^Ink4^ and p19^arf^ intermediates (26, 27), leading to cell cycle arrest and senescence (28).

## Consequences of Fetal Membrane Senescence

Fetal membranes undergo telomere-dependent aging during gestation, with the shortest telomere lengths detected post-term (~42 weeks’ gestation) (3, 29). Progressive telomere length reduction during gestation is a feature of oxidative damage to highly vulnerable G-rich repeats within the telomere DNA sequence (30). OS-induced p38MAPK activation causes ultrastructural changes characteristic of senescence, manifested by enlargement of cell and organelle (ER and mitochondria) volumes as well as damage to nuclear and plasma membranes. Unlike the phenomena of apoptosis and autophagy, clearing of senescent cells by immunological mechanisms has not been reported during pregnancy and their persistence may instigate a unique inflammatory response (31) although senescence surveillance by immune cells has been reported in other fields (32). The uterine environment senses fetal membrane aging based on its recognition of inflammatory mediators known as senescence-associated secretory proteins (SASPs) (31, 33). SASP include cytokines, chemokines, growth factors, matrix degrading enzymes, and enzymes that generate prostanoids. Many of the SASP factors are already reported to be associated with both term and preterm labor (34–42). SASPs are released from senescent fetal membrane cells and their release can be reduced by treating fetal membrane cells with p38MAPK inhibitor, suggesting the role for this stress signaler in fetal membrane aging and inflammation.

We also want to acknowledge that fetal membranes are not the only tissues that undergo aging. Multiple reports have suggested decidual aging and its association with term and preterm parturition (43–45). These reports have suggested the mechanistic role of p53, a prosenescence and apoptosis promoter, in murine decidual tissues. Although our group reports no p53-mediated senescence activation in human fetal membranes, it is likely that distinct mechanisms of senescence activation in feto-maternal compartments. All these processes may synergize to cause parturition as effecter molecules and their activation processes are likely regulated differently in various tissues. Placental aging associated with adverse pregnancy outcomes has been reviewed recently by Cox and Redman (46). Similarly, unexplained anteparturm still births have been linked to placental aging and other adverse outcomes (17, 47, 48). The rest of the review is focused on our work using fetal membrane senescence.

## Signals from Aging Fetal Membranes

Senescence-associated secretory proteins include cytokines, chemokines, angiogenic and other growth factors, matrix degrading enzymes as well as their endogenous inhibitors, cell adhesion molecules, proapoptotic receptors, and their ligands (33). Many of these factors have previously been identified in fetal and maternal tissues from term and spontaneous preterm births (31). SASPs perpetuate cellular inflammation and injury by causing the release of damage-associated molecular patterns (DAMPs) (49). Unlike inflammatory cytokines or chemokines released as specific responses to cellular stress, DAMPs are molecules with other defined intracellular signaling functions, but when leaked into the extracellular space they elicit a powerful inflammatory response (50). DAMPs from senescent fetal membrane cells include high mobility group box (HMGB)-1, a non-histone nuclear protein, HSP70, fragments of cell-free DNA, telomere repeat sequences, and uric acid (51). DAMPs are recognized by pattern recognition receptors located in the plasma membranes and on endosomes of nearby cells (52, 53). Their ubiquitous expression enables most cells to recognize and ligate DAMPs, causing inflammation, complement activation, and cell necrosis (52).

## DAMPs in a Feed Forward Loop Cause Fetal Senescence to Signal Parturition

As mentioned above, SASP markers are well reported to be associated with human parturition. Several studies have reported a higher concentration of pro-inflammatory cytokines and chemokines at term labor compared to term not in labor (54–56). Similarly, several reports have compared them between term and spontaneous preterm birth with and without preterm rupture of the membranes (34, 39, 57, 58). This surge in inflammatory markers at term labor is theorized as changes initiated by endocrine disruptions (59), vascular changes (60), and leukocyte migration and activation (61, 62). In adverse pregnancies, the inflammatory response is associated with either infectious or other risk exposures (2).

Functional role for DAMPs, specifically HMGB1 (50, 63–65), uric acid (66, 67) and cell-free fetal DNA have also been reported in term and preterm parturition (68–71). *In vitro* experiments have shown that HMGB1 released from senescent fetal cells in a feedforward loop cause increased expression of TLR2 and TLR4, cause p38MAPK-mediated senescence and inflammatory cytokine release in amnion epithelial cells. Both senescence activation and inflammatory cytokine release were inhibited by p38MAPK inhibitor SB 203580. This suggests the activation of pathway mediated by p38AMPK (72). Gomez-Lopez et al. have reported that intraperitoneal injection of HMGB1 into pregnant B6 mice leads to spontaneous preterm birth and high rate of pup mortality (73). Thus, HMGB1, a normal nuclear component, can function as a pro-parturition inflammatory mediator. Telomere fragments are released from senescent fetal cells and they are seen in high abundance in the amniotic fluid of women at term labor compared to term not in labor (74). These cell-free fetal telomere fragments are also DAMPs with immunological functions. Using cell-free telomere fragment mimics (TTAGG_2_ repeats) as a stimulant, Polettini et al. showed amnion cells undergo p38MAPK-mediated senescence and inflammatory cytokine release (8). Like in HMGB1 experiments, this effect was inhibited by p38MAPK inhibitor SB203580, confirming the role of this signaler in inducing this pathway. In addition, Polettini et al. showed that intraamniotic injection of telomere fragments into CD1 mice could cause OS, p38MAPK activation, senescence, and low birth weight and prematurity (8). Thus, multiple pieces of evidence indicate that DAMPs, such as SASPs, have a functional role in preterm and term parturition. It is unclear how these proinflammatory mediators reach from senescent fetal tissues to maternal compartments to increase an overall inflammatory load.

## SASPs and DAMPs as Exosome-Encapsulated Signals

Localized effects within the fetal membranes are insufficient to promote robust uterine contractions, but SASP and DAMP signals can be propagated across the feto-maternal interface through two different paths: (1) direct chemical diffusion to adjacent tissue layers or (2) encapsulated within exosomes, which can be transported to sites of functional activity in the myometrium, decidua, or cervix. One of the limiting steps in the former transport approach is that SASPs and DAMPs, including free HMGB1, are modified by acetylation or oxidization rendering very short half-lives in biologic fluids due to proteolytic degradation (75). By contrast, encapsulation in exosomes protects their cargo and increases the stability of potential signals by several fold. Exosomes are bioactive, spherical, cell-derived vesicles (30–100 nm) secreted during the process of exocytosis, which have been reported to increase in number as a function of duration of pregnancy (76–78). In addition to common membrane and cytosolic molecules, exosomes harbor unique, cell-specific subsets of proteins, such as HMGB1, cell-free fetal DNA, and telomere fragments. Exosomes afford a low intraluminal ambient pH, shielding contents, such as HMGB1 from oxidation, and conferring secure transport to distant sites (79). Exosomes contain molecular constituents of their cell of origin, including proteins and RNA that reflect the physiological state of the cell source and, hence, can serve as a source of representative biomarkers (80–84). Recent report by Sheller et al. has shown that exosomes from amnion epithelial cell grown under normal and OS conditions had specific markers reflective of physiologic changes (85). Similarly, in their review, Cuffe et al. found that placental OS generates biomarkers that are packaged in exosomes (86), reflecting cellular physiologic status. These placental-specific exosomes can be isolated from maternal liquid biopsies and can be used as biomarkers to determine placental function.

## Trafficking and Functional Changes Induced by Exosomes at Distant Sites

Trafficking of exosomes, delivery of cargo at specific sites and their functional role have not been well reported during pregnancy. Recent findings by Chang et al. reported expression and trafficking of placental microRNAs at the feto-maternal interface. In a model of humanized mouse, authors report expression of 160-kb human 19 miRNA cluster (C19MC) locus or lentivirally express C19MC miRNA members selectively in the placenta of mouse (87). Pregnancy caused elevated expression of C19MC miRNA in the placenta of transgenic mice that resembled C19MC miRNAs patterns in humans. The authors further report that placental miRNA traffic primarily to the maternal circulation, suggesting a paracrine mode of signaling between the fetus and the mother (87).

*In vitro* experiments have shown that oxidatively stressed fetal membrane cells secrete exosomes richer in inflammatory mediators than cells grown under control conditions (85). Exosomes are increased at term in maternal plasma samples, and particularly so during certain pregnancy complications, and are more prevalent during labor (76, 81, 88). When fetal membrane cell-derived exosomes were injected into the intraamniotic cavity of mice, they were shown to traverse across the placental layers and accumulate within maternal tissues, including the myometrium and kidneys (89). Exosomes used for these studies were isolated from human amnion cells using ultracentrifugation and size exclusion chromatography were 50–120 nm in size and exhibited tetraspanin and endosomal sorting complexes required for transport markers. In addition, amnion-derived exosomes also expressed NANOG, a stem-cell-specific marker, expressed in amnion and chorion cells but in other uterine derived cells. In our animal model study, we were able to show two key modes of propagation of exosomes: (1) diffusion of exosome through tissue layers from fetal side to maternal side of the placenta and uterus, and (2) systemic propagation of exosomes through blood to various maternal organ system. Although, we were able to determine the propagation of exosomes, we are yet to determine their functional effect on maternal side *in situ*. In human amnion cells exposed to OS *in vitro*, exosomes were secreted into the conditioned media and could be passively transferred to human myometrial cell cultures, where they fused with the uterine cells and activated host cell COX-2, connexin-43, and cytokine mRNA expression. Salomon et al. have also shown the functional effect of exosomes under different oxygen tension (90). These combined results provide a proof of concept that the fetal membranes can propagate and traffic a parturition signal to the uterus *via* exosomes.

## Exosomes as Biomarkers

Because exosome contents are specific to the derivative cell, they constitute a real-time “fingerprint” of their cell of origin (91). Thus, exosomes could potentially contribute as biomarkers of the physiologic state of fetal membrane cells during pregnancy and parturition (76, 88). Exosomes exhibit several advantages over classical soluble or “free” biomarkers present in biofluids (83, 91, 92). In particular, the stability of molecules packaged into exosomes is enhanced since they are protected from degradation *in vivo* and during storage (79).

## Aging Starts *In Utero*

In summary, our thesis provides a new concept of the mechanisms underlying human parturition. We emphasize that along with its embryonic development program, the process of organismal aging of any mammal, including the human, starts *in utero*, at the time of fertilization. *In utero* programming involves both embryonic and extraembryonic tissue longevity, ultimately creating a homeostatic, stable environment preparing the fetus for independent extrauterine existence as a neonate. However, given their strategic layering between the fetus and the maternal decidua and myometrium, it appears that the fetal membranes tissues monitor the timing of gestation and promote expulsion of the intrauterine contents *via* an outburst of sterile inflammatory mediators. These mediators are propagated vectorially, from the fetal-to-maternal direction, promoting labor-associated changes in the myometrium. Concomitant with fetal maturation, tissues of the fetal membranes age and senesce, generating exosomes that carry molecules that are transformed into a uterotonic payload (3, 6, 74). As the interaction of SASPs and DAMPs reach a threshold, myometrial activation is initiated and the birthing process is launched.

If these concepts and our preliminary data are confirmed and supported by continuing investigation, several testable hypotheses are self-evident:

1. p38MAPK inhibitors could be used to prevent or even reverse cellular damage, providing a potential therapeutic strategy to mitigate fetal membrane aging and PTB (93).
2. Exosomes expressing selective fetal membrane-specific antigens could be sampled from maternal blood as cell-specific, non-invasive “liquid biopsies” to longitudinally monitor amniochorionic membrane aging during pregnancy.
3. Given the physical characteristics of exosomes, they are suitable to a variety of mechanical separation methods that currently confound standard “OMICs” approaches to the quantification of “free” biomarkers. Moreover, enhanced stability of exosomes in biologic fluids is likely to enhance biomarker sensitivity and assay performance.
4. Custom exosomes could be used as therapeutic delivery vehicles that contain cargo that promotes uterine quiescence.

In summary, we propose a novel concept of parturition in humans mediated by paracrine factors generated by natural and physiologic aging of fetal cells through senescence. The aging trajectory of fetal membranes and placenta are likely reflections of fetal growth and maturation. Aging, an inflammatory condition, generate inflammatory mediators, including DAMPs and well characterized uterotonins and propagate them to various feto-maternal tissues through exosomes. These communication channels reflect physiologic status of their cells of origin. Thus, exosome cargo contents reflect pregnancy status and, therefore, can function as potential biomarkers. Ongoing research has successfully isolated placental-derived exosomes from maternal plasma during normal and abnormal pregnancies (81). Future research in this area is expected to provide novel insights into fetal signaling during pregnancy and parturition.

## Author Contributions

RM, SM, and RT conceived the idea and drafted the manuscript.

## Conflict of Interest Statement

The authors declare that the research was conducted in the absence of any commercial or financial relationships that could be construed as a potential conflict of interest. The reviewer, GR, and handling editor declared their shared affiliation, and the handling editor states that the process nevertheless met the standards of a fair and objective review.
